# Molecular and Cellular Pathways Contributing to Joint Damage in Rheumatoid Arthritis

**DOI:** 10.1155/2020/3830212

**Published:** 2020-03-17

**Authors:** Qinghua Fang, Chun Zhou, Kutty Selva Nandakumar

**Affiliations:** SMU-KI United Medical Inflammation Center, School of Pharmaceutical Sciences, Southern Medical University, Guangzhou, China

## Abstract

Rheumatoid arthritis is a chronic autoimmune syndrome associated with several genetic, epigenetic, and environmental factors affecting the articular joints contributing to cartilage and bone damage. Although etiology of this disease is not clear, several immune pathways, involving immune (T cells, B cells, dendritic cells, macrophages, and neutrophils) and nonimmune (fibroblasts and chondrocytes) cells, participate in the secretion of many proinflammatory cytokines, chemokines, proteases (MMPs, ADAMTS), and other matrix lysing enzymes that could disturb the immune balance leading to cartilage and bone damage. The presence of autoantibodies preceding the clinical onset of arthritis and the induction of bone erosion early in the disease course clearly suggest that initiation events damaging the cartilage and bone start very early during the autoimmune phase of the arthritis development. During this process, several signaling molecules (RANKL-RANK, NF-*κ*B, MAPK, NFATc1, and Src kinase) are activated in the osteoclasts, cells responsible for bone resorption. Hence, comprehensive knowledge on pathogenesis is a prerequisite for prevention and development of targeted clinical treatment for RA patients that can restore the immune balance improving clinical therapy.

## 1. Introduction

**Figure 1:**
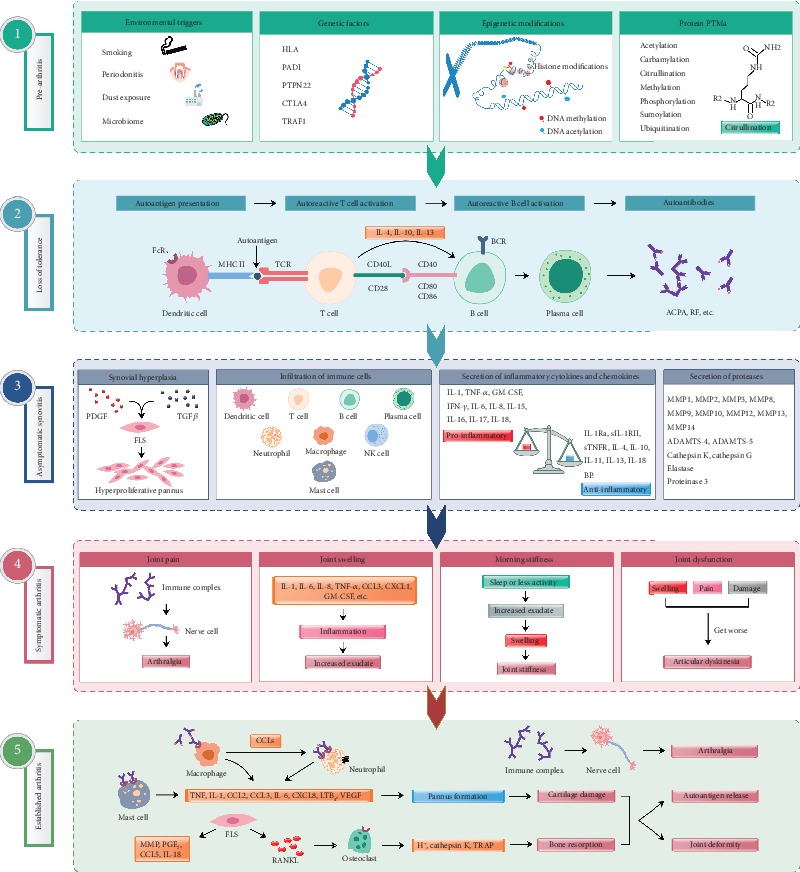
Different phases in RA pathogenesis. (1) Genetic, epigenetic, and environmental factors contribute to arthritis progression. Multiple environmental risk factors (for example, smoking, pollutants, or microbes), when come in contact with the mucosal sites, are most likely responsible for causing local inflammatory events and immune system activation inducing epigenetic modifications and protein posttranslational modifications (PTMs) [59], before crossing the threshold to trigger disease in genetically vulnerable people. (2) Dendritic cells presenting altered self or related peptides to T cells (breakdown of tolerance mechanisms) leads to the activation of T and B cells effectuating synthesis of cytokines and autoantibodies. Progressively, these autoantibodies are produced more and more, which recognize several neoepitopes by the process of epitope spreading, and gets overt during the onset of the clinical disease [1]. (3) Disease development involves autoimmune responses against both posttranslationally modified and unmodified self-antigens, which starts many years before the subclinical synovitis and appearance of clinical symptoms [60]. (4) Autoantibodies induced during this preclinical phase can also be responsible for bone erosion and pain. Before the onset of inflammation, these alterations could reduce overall functions of the joints. After the autoantibodies start binding to different epitopes and form immune complexes, inflammation in the synovium and development of arthritis ensue. (5) Antibody-induced cartilage and bone changes, if minor, resolve without any considerable damages. However, if left untreated or in the presence of continuous external stimuli, these changes can give rise to chronic inflammation, joint destruction, and disability [59]. Arthritis is associated with both local as well as systemic pathological manifestations. ACPA: anticitrullinated protein antibody; ADAMTS: a disintegrin and metalloproteinase with thrombospondin motifs; BCR: B cell receptor; CCL: c-c motif chemokine ligand; CXCL: C-X-C motif chemokine ligand; CTLA4: cytotoxic T-lymphocyte-associated protein 4; FcR: Fc receptor; FLS: fibroblast-like synoviocytes; GM-CSF: granulocyte-macrophage colony-stimulating factor; HLA: human leukocyte antigen; IFN-*γ*: interferon gamma; IL: interleukin; IL-1Ra: interleukin-1 receptor antagonist; IL-18BP: interleukin-18-binding protein; LTB_4_: leukotriene B4; MMP: matrix metalloproteinase; MHC II: major histocompatibility complex class II; NK cell: natural killer cell; PADI: peptidyl arginine deiminase; PDGF: platelet-derived growth factor; PGE_2_: prostaglandin E2; PTPN22: protein tyrosine phosphatase nonreceptor type 22; RANKL: receptor activator of nuclear factor kappa B ligand; RF: rheumatoid factor; sIL-1RII: soluble interleukin 1 receptor II; sTNFR: soluble tumor necrosis factor receptors; TCR: T cell receptor; TGF*β*: transforming growth factor *β*; TNF-*α*: tumor necrosis factor *α*; TRAF1: TNF receptor-associated factor 1; VEGF: vascular endothelial growth factor.

Rheumatoid arthritis (RA) is a chronic, inflammatory syndrome comprised of various disease phenotypes. RA is characterized by aggressive synovial hyperplasia causing destruction of articular joints. A combination of genetic, epigenetic, and environmental factors is responsible for the onset and development of RA. An array of susceptible genes (human leukocyte antigen (HLA) class II and more than 100 susceptibility loci including PTPN22, PADI4, TRAF1, and CTLA4), nongenetic factors (sex hormones, smoking, periodontal infection, and microbiota), immune (macrophages, dendritic cells, mast cells, neutrophils, T cells, and B cells) and nonimmune (fibroblasts and chondrocytes) cells, and inflammatory mediators (autoantibodies, cytokines, chemokines, and proteases) are collectively involved in the inflammatory processes targeting the cartilage and bone effectuating functional loss of joints (Figure 1) [1].

The synovium is one of the major target tissues in RA [2]. During joint inflammation, macrophage-like synoviocytes (MLS) and fibroblast-like synoviocytes (FLS) proliferate to form the pannus, which invades and destroys the cartilage. These cells are the major sources of factors that can promote inflammation and joint destruction. Autoantibodies contribute to the inflammatory process by acting as the mediator of joint inflammation and bone erosion [3]. 50–80% of RA patients have autoantibodies depending on the duration of the disease. Autoantibodies can initiate inflammatory effector pathways, which affect chondrocytes and the cartilage causing release of extracellular matrix (ECM) components. In this context, glycosylation of autoantibodies is crucial. Decreased IgG-Fc sialylation is associated with RA and osteoclastogenesis, while an increase in sialylation decreased inflammatory bone loss [4]. Bone erosion and loss of physical function in arthritis begin early and progress along with disease severity. The main triggers of bone erosion are an inflamed synovium, proinflammatory cytokines, autoantibodies, and receptor activator of nuclear factor *κ*B ligand (RANKL). Breakdown of self-tolerance causes activation of immune and nonimmune cells resulting in the production of inflammatory mediators. Fibroblasts expressing RANKL together with macrophage colony-stimulating factor (M-CSF) promote differentiation of preosteoclasts into bone-resorbing osteoclasts; this process is initiated at the junction of the cartilage and bone. Targeting T and B cells, proinflammatory mediators, signaling molecules, and synovium-specific targets are pursued as new treatment options [5]. In this review, we discuss about disease pathways contributing to cartilage and bone damage to facilitate research on developing targeted drugs.

## 2. T Cells

Increasing evidences demonstrate that RA development results due to an imbalance between CD4^+^ T cell subsets [6, 7]. Under physiological conditions, T cells are tolerant toward self-antigens [8, 9]. The presence of T cells in the inflamed synovium, association of arthritis with HLA loci, and transfer of disease using T cells in rodent models suggest the importance of T cells in arthritis pathogenesis. However, difficulty in identifying consistent T cell effector mechanisms and not so convincing results from T cell-targeted therapies question this view. Currently, CD4^+^ T cells reactive with citrulline were identified and the detection rate was high during the first 5 years after RA diagnosis. Although this finding provides a theoretical basis for the correlation between citrulline-specific CD4^+^ T cells and RA, the mechanisms of T cell activation and its role in promoting joint inflammation need further investigation. Recent studies identified a defective glycolytic process present in T cells from RA patients, which causes glucose to be diverted into the pentose phosphate pathway, driving the accumulation of NADPH and ROS consumption. With an excess of reducing equivalents, T cells are unable to activate the relevant redox kinase, which enabled bypassing the regulatory checkpoint of the G2/M cell cycle that is conducive for their excessive proliferation [10].

Citrulline-specific CD4^+^ T cells of Th1 memory phenotype are higher in RA patients [11]. Upon stimulation with many cytokines, CD4^+^ T cells differentiate into Th1, Th2, and Th17 cells secreting different cytokines, while an excessive production of Th17 cells associates with disease severity in many autoimmune diseases. Th17 cells can also mediate osteoclast activation and synovial neovascularization causing bone erosion. Overexpression of Th17-specific transcription factor, retinoic acid-related orphan receptor (ROR*γ*t), not only induced a high expression of chemokine receptor 6 (CCR6) but also promoted CD4^+^ T cell migration into the affected joints through the CCR6-specific chemokine ligand 20 (CCL20) pathway [7].

On the other hand, Treg cells by secreting IL-10 and TGF-*β* maintain lymphocyte homeostasis and tolerance. When a RA mouse model was administered with sialic acid-binding Ig-like lectin-9, Th17 cell differentiation was reduced and Treg cells proliferated, which attenuated joint inflammation and bone damage. In this context, Foxp3 plays a crucial role by affecting the glycolysis and metabolism of Treg cells through the phosphatidylinositol 3-kinase/protein kinase B/rapamycin target protein (PI3K/Akt/mTOR) signaling pathway [12].

It has been suggested that PTPN22 encoding a tyrosine phosphatase contributes to the immune tolerance by limiting the signaling events after recognition of autoantigens and weak agonistic antigens by T cell antigen receptors (TCRs) of naive and effector T cells, while not hindering the response to foreign antigens [13]. This observation suggests that inactivation of the PTPN22 allele can amplify the effector or memory T cells, which possibly could enhance the development of an autoimmune disease. More importantly, the PTPN22-related alleles have a stronger interaction with arthritis-susceptible HLA-DR alleles. Therefore, an in-depth functional study of PTPN22 gene polymorphisms in arthritis development may further improve our understanding of RA pathogenesis.

## 3. Dendritic Cells

During homeostasis, dendritic cells (DCs) are involved in the maintenance of immune regulation and tolerance. However, in RA by presenting self-peptides, they trigger differentiation and activation of the auto-reactive T cells as well as innate immune effector functions [14]. In RA patients, increased numbers of DCs are present in the synovial fluid and tissues. Interestingly, tolerogenic DCs (TolDCs) can be generated by genetic and pharmacological modifications or by using cytokines in an antigen-specific manner. Induction of such immune tolerance mechanisms is a promising approach to treat or prevent autoimmune disorders. Many application methods to achieve antigen-specific therapy were reviewed recently [15]. Autologous self-antigen-loaded TolDCs are capable of deleting or reprogramming auto-reactive T cells and were used for the treatment of experimental arthritis with more promising results [16].

## 4. B Cells and Autoantibodies

Importance of B cells in the pathogenesis of RA has been studied and discussed extensively. Initially, the role of B cells in arthritis was appreciated mainly in terms of autoantibodies because of their importance in clinical diagnosis and prognosis and also as inflammatory mediators. However, B cells can also contribute to disease pathogenesis through antibody-independent mechanisms [17] including antigen presentation, modulation of T and dendritic cell functions, and production of proinflammatory and regulatory cytokines, facilitating the tertiary lymphoid tissue formation in target organs and possibly tissue repair as well. In recent years, the clinical efficiency of B cell-targeted therapies has revealed the pathogenic properties of B cells clearly in several inflammation-dependent diseases [18]. Recent studies have provided insights into the enrichment of memory B cell subsets distinguished by the expression of Fc-like receptor 4 (FcRL4) in the joints and mucosa-associated lymphoid tissues of RA patients. Interestingly, antibodies produced from FcRL4^+^ B cells have high binding capacity to citrullinated autoantigens [19].

**Figure 2:**
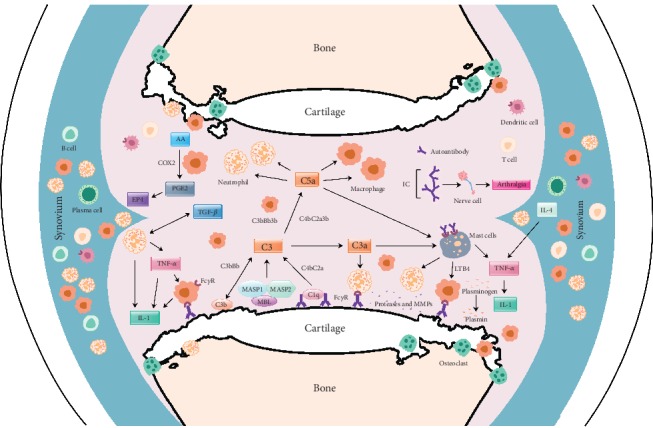
Likely interactions of molecules and factors in the antibody mediated joint inflammation. Upon binding to joint antigens or deposited as immune complexes on the cartilage surface, autoantibodies initiate inflammation-dependent and inflammation-independent activities, which culminate in the direct damage to the cartilage and bone. Activation of complement cascades by autoantibodies leads to the release of anaphylatoxins (C3a, C5a), attracting FcR-bearing immune cells to the inflammation foci, which in turn get more activated and secrete cytokines that can further activate resident nonimmune cells in the joint. All these cells in the inflamed joint secrete more inflammatory mediators and extracellular matrix lysing enzymes that could destroy the cartilage and bone. AA: arachidonic acid; C1q, C2a, C3, C3a, C4b and C5a and B (factor B): complement components; CCL3: chemokine (C-C motif) ligand 3; COX2: cyclooxygenase-2; EP4: prostaglandin receptor; MASP: mannose-associated serine protease; MBL: mannose-binding lectin; IC: immune complex; IL: interleukin; LTB4: leukotriene B4; Fc*γ*R: Fc gamma receptors; PGE2: prostaglandin E2; TGF: transforming growth factor; TNF: tumor necrosis factor.

Autoantibodies are highly prevalent and detectable in RA patients' sera several years before the clinical symptoms appear [20]. During the time of disease onset, epitope spreading [21], avidity maturation [22] and proinflammatory IgG-Fc glycosylation phenotype [23] of ACPAs were found to occur. Interestingly, the presence of desialylated ACPAs is more during active joint inflammation and transfer of sialic acid-enriched antibodies attenuated experimental arthritis [24]. Alterations in N-glycome induce Fc conformational changes that have direct influence on antibody effector and immunoregulatory functions. Furthermore, germ line-encoded antibodies were identified to be important in experimental arthritis and self-antigen-specific B cells were neither deleted nor anergized [25]. Autoantibodies after binding to their target antigens trigger downstream inflammatory cascades either directly or in the form of immune complexes [26]. At this effector phase of arthritis, activation of different pathways of complement [27, 28] and Fc*γ*R-bearing immune cells contributes to cartilage destruction either directly or by promoting the secretion of inflammatory cytokines and matrix lysing enzymes (Figure 2) [29]. At the same time, antibodies binding to collagen type II (CII) can also induce target damage independent of inflammatory mediators or cells [30]. These antibodies disrupt the integrity of the cartilage matrix by promoting impaired cartilage formation, inhibiting cartilage fibril generation, and disassembling CII fibrils in the ECM. In addition, anti-CII antibodies induced pain prior to and after the appearance of arthritis symptoms and involved in immune complex-mediated activation of neurons [31]. Upon passive transfer, purified anti-CII antibodies from RA patients induced arthritis in naive mice [32], which demonstrated their pathogenicity. Unlike anti-CII antibodies, ACPAs might be nonpathogenic [33]. Conversely, ACPAs were shown to mediate osteoclastogenesis [34] and be responsible for bone loss prior to the onset of clinical arthritis [35]; ACPAs induced pain [36] and FLS migration through activation of phosphoinositide 3-kinase [37]. Immune complexes or ACPAs from RA patients induced TNF-*α* production in peripheral blood mononuclear cells and macrophages [38]. Furthermore, ACPAs were suggested to be agonists for a receptor-mediated response, but this notion is still controversial [39]. Hence, more studies are needed to understand all the possible roles of ACPAs in RA.

## 5. Macrophages

Macrophages are central in perpetuating arthritis development by stimulating neovascularization, clearing apoptotic immune cells, and promoting the proliferation of fibroblasts and secretion of proteases. Based on the expression of surface molecules, cytokine secretion, and arginine metabolism, macrophages are classified into pro- (M1) and anti-inflammatory (M2) phenotypes [40]. The synovial macrophages of RA patients are of M1 phenotype, which highly express proinflammatory proteins PHD3, CCR2, MMP12, and TNF-*α* with a concomitant low expression M2-type polarization markers [41]. In addition, the level of synuclein A (activin A) encoded by the proinflammatory INHBA gene is significantly elevated in RA patients produced by activated macrophages that can mediate M1-type polarization [42]. Such polarized M1 macrophages secrete a large number of proinflammatory cytokines (IFN-*γ*, TNF-*α*, IL-1, and IL-6), chemokines (CCL5, CXCL-1, and CXCL-10), and various matrix lysing enzymes, which in turn activate fibroblasts and osteoclasts; aid in the recruitment of neutrophils, monocytes, and lymphocytes; and trigger a series of inflammatory reactions that accelerate inflammation and cause destruction to the articular cartilage. In RA, such a high activation of macrophages increases the expression of toll-like receptors (TLR2, TLR3, TLR4, and TLR7) and promotes the synovial inflammation and cartilage destruction by producing enzymes, cytokines, and other inflammatory factors [43]. In addition, autophagy of macrophages plays an essential role in the pathogenesis of RA [44], which can increase the number of osteoclasts contributing to enhanced bone resorption activity.

## 6. Neutrophils

In RA, neutrophils may alter immune regulation by increasing their cell survival and mobility, having anomalous inflammatory activity, increasing oxidative stress, releasing of neutrophil extracellular traps (NETs), and also by interacting with resident FLS in the synovium to promote inflammatory and antigen-presenting phenotype [45]. High levels of NETs are present in the serum, synovial tissue, rheumatoid nodules, and skin of ACPA^+^ RA patients. The formation of NETs requires two major biochemical activities. First, the inactivation of the PTPN22 enzyme is considered to be necessary for the production of NETs, which may be involved in the removal of the nuclear envelope and NET components [46]. Second, NETs are composed of DNA and histones, which can be acted upon by peptidyl arginine deiminase type IV (PADI4) causing citrullination. This process prevents histone methylation and transcription leading to chromatin depolymerization, a central event in the NET formation [46]. In addition, NETs contain PAD deposits that promote the formation of citrulline products. Interestingly, the level of NETs in the plasma are highly specific (92%) and sensitive (91%) during diagnosis of early RA patients.

## 7. Fibroblasts

Normal FLS in the synovial intimal lining layer has important functions in the maintenance of joint homeostasis by secreting hyaluronan, lubricin, and plasminogen activator; controlling synovial fluid volume and normal inflammatory responses; and regulating leukocyte trafficking and in the maintenance of the joint capsule. In arthritis, FLS are hyperproliferative and an impaired apoptosis could also promote accumulation of FLS in the joints. In RA, FLS produces cytokines and proteases, apart from acquiring an aggressive, tumor-like phenotype because of transcriptional mechanisms of imprinting and epigenetic changes, which could mediate cartilage destruction and drive joint inflammation [47]. IL-17 is one of the crucial factors in transforming FLS into an invasive RA-FLS type and may directly assist in FLS-mediated progression of RA by significantly increasing its activation, migration, and invasive potential. RA-FLS also secretes many proangiogenic factors like fibroblast growth factor, vascular endothelial growth factor (VEGF), hypoxia-inducible factors (HIFs), and IL-18, which promote new blood vessel formation, pannus growth, and inflammation.

## 8. Chondrocytes

Chondrocytes are unique to the articular cartilage, which maintain an equilibrium between synthesis and breakdown of extracellular matrix under physiological conditions. A chondrocyte secretome contains extracellular matrix proteins, cytokines, growth factors, enzymes, and their inhibitors as well as many other protein components having different target specificities [48]. Cytokines trigger chondrocytes to release more cytokines and matrix metalloproteinases (MMPs) that can degrade the cartilage and also inhibit generation of tissue inhibitors of metalloproteinases (TIMPs). IL-1*β* released during inflammation can increase the catabolic activities of chondrocytes by inhibiting the spontaneous calcium signaling as well as in altering signaling in the cell cycle and Rho GTPases present within the chondrocytes. Dependent on the release of proinflammatory cytokines from the synovium, chondrocytes are activated to participate in cartilage damage. At the same time, they could also act as the source of proinflammatory cytokines, which in turn increases catabolic events in the cartilage while suppressing anabolic tissue repair and remodeling processes.

## 9. Role of Cytokines

Cytokines are involved in many inflammatory events related with the regulation of inflammation, autoimmune responses, synovitis, and articular joint destruction. Many crucial cytokines (IL-1, IL-6, IL-10, IL-12, IL-15, IL-17, IL-18, TNF-*α*, TGF*β*, IL-23, etc.) [49] (Table 1) and all the four family of chemokines (CXC, CC, C, and CX_3_C) [50] (Table 2) are contributing to the joint inflammation. Successful amelioration of signs and symptoms of arthritis in patients with TNF-*α*-neutralizing agents has transformed RA treatment strategies quite significantly, which facilitated further research to target other inflammatory cytokines like IL-1, IL-6, and IL-17. However, targeting cytokines should be done with caution because they are pleiotropic, redundant, and multifunctional apart from having antagonistic and synergistic functions between them. Many RA patients are still reported to be refractory to anti-TNF-*α* therapy and TNF inhibitors were found to be more effective in clinical trials than in daily clinical practice. Moreover, anti-inflammatory cytokines also can promote joint inflammation [51]. Pro- and anti-inflammatory cytokines and chemokines involved in joint inflammation/resolution, their cellular sources, target cells, and major functions are summarized in Tables 1 and 2, respectively.

## 10. MMPs, ADAMTS, and TIMPs

Several proteinases like MMPs, ADAMTS (a disintegrin and metalloproteinase with thrombospondin motifs), neutrophil elastase, and cathepsins (G and B) can damage the cartilage directly. Depletion of proteoglycans from articular cartilage is an initial event in RA development leading to the degradation of the collagen fibrils. It is of interest to note that CII-reactive monoclonal antibodies upon passive transfer induced a significant amount of proteoglycan depletion within 72 hours [30]. Many MMPs (MMP 1-3, MMP 7-9, MMP-13, and MT1-MMP) preferentially split the bond between Asn^341^-Phe^342^ of aggrecan. Conversely, ADAMTS1, ADAMTS4, and ADAMTS5 cleave the Glu^373^-Ala^374^ bond in addition to other sites in the G2-G3 domains of proteoglycans. Thus, both the MMP and ADAMTS enzymes contribute to aggrecan degradation during arthritis development. TIMPs are endogenous blockers of MMPs and regulators of matrix turnover, tissue reorganization, and cellular activity. Sources, targets, and receptors/ligands and major functions of MMPs, ADAMTS, and TIMPs involved in arthritis pathogenesis are summarized in Table 3.

## 11. Signaling Pathways Affecting Bone Destruction

**Figure 3:**
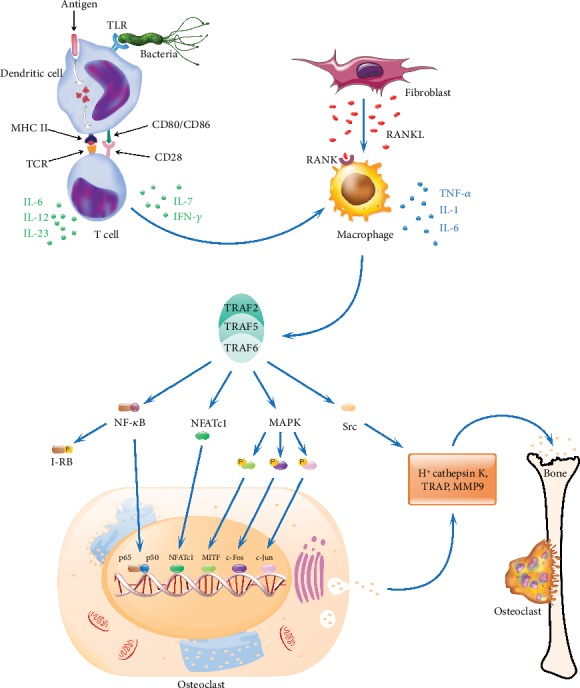
Signaling pathways in osteoclast activation. During RA pathogenesis, antigen-presenting cells after uptake of an autoantigen or pathogenic molecules process and present antigenic determinants on their cell surface in conjunction with arthritis-permissible HLA/MHC class II molecules, which activate differentiation of T cells into different subphenotypes. The activated T cells secrete various cytokines like IL-6, IL-7, IL-10, IL-12, IL-17, IL-23, and IFN-*γ*. These cytokines modulate macrophages to secrete various pro- and/or anti-inflammatory cytokines and other inflammatory mediators. Upon exposure to the inflammatory cytokines, fibroblast-like synoviocytes express RANKL, which binds with its receptor (RANK) present on the cell surface of activated macrophages initiating the RANK/RANKL pathway through TRAF 2, 5, and 6 proteins, which leads to the activation of downstream NF-*κ*B, MAPK, NFATc1, and Src signaling cascades. These factors after translocation initiate the expression of genes like TRAP, CtsK, and MMP-9 in the nucleus, which promote osteoclastogenesis and bone resorption. TLR: toll-like receptor; TCR: T cell receptor; CtsK: cathepsin K; I-*κ*B: inhibitor of the NF-*κ*B transcription factor; IL: interleukin; IFN-*γ*: interferon gamma; MAPK: mitogen-activated protein kinase; MHC II: major histocompatibility complex II; MITF: microphthalmia-associated transcription factor; MMP 9: matrix metalloproteinase 9; NFATc1: nuclear factor of activated T cells, calcineurin-dependent 1; NF-*κ*B: nuclear factor kappa B; p50 and p65: REL-associated proteins (also called NF-*κ*B1 and RelA) involved in NF-*κ*B heterodimer formation and nuclear translocation and activation; RANK: receptor activator of nuclear factor-*κ*B; RANKL: receptor activator of nuclear factor *κ*B ligand; Src: intracellular non-receptor tyrosine kinase; TRAF: TNF receptor-associated factor; TRAP: tartrate-resistant acid phosphatase. c-Jun and c-Fos form the early response transcription factor, AP-1.

Bone erosion starts during the early phase of arthritis development causing deformity of the articular joints, which affects quality of patients' life. Molecular mechanisms underlying differentiation and activation of bone-eroding cells, osteoclasts, are well documented, and several signaling pathways are contributing to osteoclast maturation and activation causing joint destruction (Figure 3).

## 12. RANKL/RANK Pathway

RANKL (also called TNFSF11, OPGL, TRANCE, and ODF) and its receptor RANK are indispensable regulators of bone repair and remodeling processes. Several hormones and cytokines induce RANKL production in osteoblasts and synovial fibroblasts. After binding with RANK, RANKL triggers the recruitment of an adaptor molecule TRAF-6 resulting in the activation of signaling molecules like NF-*κ*B, c-Jun N-terminal kinase (JNK), AKT/PKB, ERK, Src, and p38 mitogen-activated protein (MAP) kinases and the transcription factor, and nuclear factor of activated T cells, calcineurin-dependent 1 (NFATc1) [52]. Hence, the RANKL/RANK signaling pathway is a potential therapeutic target in osteolytic diseases. Denosumab (RANKL-specific human monoclonal antibody) is currently used for treating osteoporosis, osteosarcoma, multiple myeloma, and bone metastasis [53]. Although denosumab is highly specific to RANKL and has a good effect on bones, safety concerns still exist. On the other hand, the RANKL/RANK pathway is having important functions in osteoblasts as well. Vesicular RANK, secretion product of matured osteoclasts, by binding to osteoblast-derived RANKL facilitates bone formation by initiating RANKL reverse signaling leading to the activation of Runt-related transcription factor 2 (Runx2) [54].

## 13. NF-*κ*B Signaling Pathway

Initiation of the RANKL/RANK pathway causes NF-*κ*B activation, which contributes to osteoclast differentiation. After NF-*κ*B stimulation, several TNF-receptor- (TNFR-) related factors associate with the cytoplasmic domain of RANK. Among them, TRAF-6 is indispensable for osteoclast formation and activation [55], while NF-*κ*B p50 and p52 subunits modulate RANKL and TNF-*α*-induced differentiation of osteoclast precursors. Mice deficient in p50 and p52 proteins are osteopetrotic. NF-*κ*B-activating upstream catalytic (IKK-*α* and IKK-*β*) and noncatalytic (IKK-*γ* also known as NEMO) subunits of I*κ*B kinase are also crucial in the generation of osteoclasts. RelB is the NF-*κ*B-inducing kinase (NIK) downstream subunit, which is also responsible for osteoclast differentiation.

## 14. MAPKs

Mitogen-activated protein kinase (MAPK) lineage consists of p38-MAP kinases (p38-MAPK *α*, *β*, *γ*, and *δ* isoforms), c-Jun N-terminal kinases (JNK1-3), and extracellular signal-regulated kinases (ERK1-2). RANKL stimulation activates many of these kinases, which regulate different cellular responses. When a specific inhibitor (SB203580) or a natural product from teasel for p38-MAPK*α* and *β* was used, significant inhibition of osteoclast formation but not its functions was observed. RANKL-activated p38-MAPKs can directly phosphorylate STAT1 and regulate the expression of target genes. In addition, JNKs and their upstream kinase MKK7 are also involved in osteoclastogenesis. However, neither JNK1 nor JNK2 deficiency led to significant bone defects. Mice deficient in both JNK1 and JNK2 have embryonic lethality during midgestation [56]; hence, a conditional knock-out in the bone marrow might address the importance of JNKs in osteoclastogenesis. AP-1 and related genes (c-Jun, JunB, c-Fos, and Fra but not JunD), controlled by JNKs, are also crucial for osteoclast differentiation and maturation. ERK is another MAPK subunit getting activated upon RANKL stimulation, which regulates the survival and differentiation of both osteoclasts and osteoblasts. However, several receptor systems in many organs operate via NF-*κ*B and MAPK pathways; hence, targeting these pathways might not be optimal for treating bone damage.

## 15. NFATc1

Stimulating with RANKL leads to the induction of several genes mediating osteoclast differentiation and function, including cellular fusion, polarization, and secretion of acid hydrolases. Some of these genes are transcribed by another key factor, NFATc1 [57], which is a downstream target of RANKL. NFATc1 expression causes osteoclast differentiation and induction of osteoclast-related genes including TRAP, cathepsin K, and calcitonin receptors in association with c-Fos. NFATc1-deficient cells are defective in osteoclastogenesis. In the absence of RANKL, overexpression of NFATc1 induced osteoclast precursor cell differentiation into TRAP^+^ osteoclast-like cells. Moreover, c-Jun signaling is also critical in the regulation of NFATc1 activation. Transgenic mice specifically expressing dominant-negative form of c-Jun in the osteoclasts exhibit severe osteopetrosis. Thus, NFATc1 acts as an important mediator in coupling RANK signaling events to osteoclast differentiation.

## 16. Src Kinase

Src is a protein-tyrosine kinase involved in the cell development, division, relocation, and survival. The resorption process of osteoclasts depends on their attachment and movement on the surface of the bones to form a sealing zone [58]. Targeted interference in c-Src gene expression led to the development of osteopetrosis, and it is a critical factor in RANKL-induced activation of protein tyrosine kinase 2 (Pyk2) and *α*v*β*3 integrin assembly, which is essential for the adhesion and skeleton organization. Binding of RANK and RANKL meditates the recruitment of TRAF-6 and c-Src. Subsequently, TRAF-6 enhances c-Src activation causing phosphorylation of signaling molecules like E3 ubiquitin-protein ligase, and Cbl. Src complexes with Pyk2, Cbl, and ADAP (adhesion and degranulation promoting adaptor protein, also called SLAP-130 or Fyb). Phosphorylation of these signaling molecules is a prerequisite for integrin-mediated osteoclast functions. Hence, targeting c-Src might be a viable future treatment strategy for osteoporosis and higher bone resorption observed in RA patients.

## 17. Conclusions

Interplay between multiple factors engender aberrations in immune recognition and activation causing initiation of molecular pathways targeting cartilage and bone. Various immune and nonimmune cells are crucial during this process. Resident and infiltrating cells in the joints proliferate and secrete proinflammatory cytokines, chemokines, and matrix lysing enzymes that could destroy the joints leading to functional loss. Moreover, different signaling cascades are activated during osteoclast activation and differentiation that are involved in the bone resorption activity. Hence, targeting a single effector molecule is insufficient to block cartilage and bone damage in arthritis. Since RA is an immune-mediated disorder, therapeutics restoring immune balance certainly can improve clinical therapy.

## Figures and Tables

**Table 1:** Pro- and anti-inflammatory cytokines in RA.

Cytokines	Sources	Affected cells	Types	Major functions
Proinflammatory				
TNF-*α*	Fibroblast, endothelial cell, and macrophage	Synoviocyte, chondrocyte, osteoclast, and endothelial cell	None	Promotes pannus tissue formation; synovial inflammation; synthesis of MMPs, PGE2, and collagenase; vasospasm, joint destruction; osteoclastogenesis; and bone resorption [61].
IFN-*γ*	NK cell, T cell, macrophage, B cell, and dendritic cell	T cell, B cell, macrophage, endothelial cell, and APC	None	Activates antigen-presenting cells, upregulates the expression of MIC molecules on monocytes, and aggravates the local inflammatory responses in RA.
IL-1	Fibroblast, macrophage, endothelial cell, synovial cell, and chondrocyte	Synovial cell, macrophage, neutrophil, B cell, T cell, osteoclast, synaptic cell, and chondrocyte	IL-1*α*, IL-1*β*, IL-18, IL-1, F5-10, and IL-33	Promotes TNF-*α* and IL-6 production, vasospasm, synaptic cell and chondrocyte synthesis, release of PGE2, secretion of MMPs, proliferation of synovial cells, and osteoclast differentiation and activity. Acts synergistically with TNF-*α* to damage the cartilage.
IL-2	T cell, monocyte, dendritic cell, and synovial cell	T cell, B cell, macrophage, and NK cell	None	Activates and maintains T cell differentiation and proliferation, mediates phosphorylation of STAT5, and triggers active transcription in Treg cells [12].
IL-6	Osteoblast, stromal cell, T cell, B cell, fibroblast, endothelial cell, monocyte, and keratinocyte	Th17 cell, B cell, osteoclast, macrophage, neutrophil, and synoviocyte	None	Regulates osteoclast formation and bone resorption, promotes proliferation and differentiation of activated B cells, enhances the effects of IL-1 and TNF-*α*, maintains differentiation and homeostasis of Th17 cells, triggers synthesis of acute-phase proteins, and induces synovial neovascularization.
IL-8	Monocyte, FLS, macrophage, synoviocyte, synovial lining cell, and endothelial cell	Neutrophil, NK cell, T cell, synovial cell, chondrocyte, and macrophage	None	Chemotactic and activation factor for neutrophils, stimulates NK and T cells to express *γ*-interferon and FasL, respectively, promotes apoptosis of T and B cells, stimulates synovial cells, and chondrocytes to produce NO, PGE2, MMPs, TNF-*α*, and IL-6.
IL-15	Macrophage, fibroblast, endothelial cell, dendritic cell, myocyte, epithelial cell, and astrocyte	CD4^+^ and CD8^+^ T cell, monocyte, macrophage, and NK cell	None	Promotes the accumulation and activation of T cells into the joints; secretion of other cytokines (TNF-*α*), adhesion molecules, proteases, and monocyte chemotactic factors (MCP-1 and IL-8); and expression of IL-17 and MMPs (MMP-1 and MMP3). It is associated with bone destruction and secretion of RF in RA [62].
IL-17	CD4^+^ Th17 cell, NK cell, neutrophil, eosinophil, monocyte, and mast cell	T cell, fibroblast, synovial cell, macrophage, neutrophil, osteoclast, synaptic cell, chondrocyte, endothelial cell, and epithelial cell	IL-17A, IL-17B, IL-17C, IL-17D, IL-17E, and IL-17F	By binding to IL-17RA/RC receptors, stimulates production of TNF-*α*, IL-1*β*, IL-6, IL-8, IL-23, chemokines, nitric oxide, prostaglandins, GM-CSF, and MMPs; promotes RANKL expression, NF-*κ*B activation, and vasospasm formation; activates osteoblasts and osteoclasts by regulating the RANKL/RANK/OPG signaling pathway; and inhibits the synthesis of proteoglycans and collagen [63].
IL-18	Macrophage, synoviocyte	Th1 cell, chondrocyte, synovial cell	None	Promotes expression of mononuclear factors and TNF-*α*.
IL-23	Macrophage, dendritic cell, and B cell,	T cell, dendritic cell, and macrophage	None	Induces formation and differentiation of Th17 cells, differentiation of osteoclasts, e production of IL-6, TNF-*α*, IL-1*β*, and chemokines (CXCL-1, GCP-2, and CXCL-8).

Anti-inflammatory				
IFN-*α*	Leukocyte, dendritic cell	T cell, monocyte, macrophage, NK cell, synoviocyte, and B cell	None	Inhibits synthesis of collagen, expression of MHC antigens, proliferation of B cells, release of prostaglandins from monocytes, and bone resorption and disrupts the balance between collagen and fibronectin.
IFN-*β*	FLS, macrophage, dendritic cell, and osteoclast	T cell, dendritic cell, macrophage, NK cell, fibroblast, and osteoclast	None	Reduces the production of TNF-*α*, IL-1*β*, and IL-6 and increases the secretion of IL-1R*α* and IL-10, modulates signaling molecules involved in the NF-*κ*B pathway, and inhibits osteoclastogenesis and the negative feedback pathway of c-fos [64].
IL-1Ra	Monocyte, macrophage, neutrophil, epithelial cell, and fibroblast	Monocyte, macrophage, lymphocyte, epithelial cell, and fibroblast	None	Abolishes IL-1 activation.
IL-4	Th2 cell, mast cell, basophil, and dendritic cell	B cell, T cell, macrophage, dendritic cell, chondrocyte, FLS, osteoclast, and APC	None	Inhibits the production of MMPs, IL-1*β*, IL-6, IL-8, IL-12, IL-17, and TNF-*α* and promotes the production of IL-1R*α* and soluble TNF receptors, directs B cells to produce antibodies like lgG1 and lgE, and promotes the expansion of Th2 cells and adhesion of macrophages to vascular endothelial cells [65].
IL-10	Th2 cell, B cell, macrophage, mast cell, monocyte, synovial cell, and chondrocyte	B cell, Th1 cell, macrophage, APC, and monocyte	None	Inhibits the expression of MHC class II antigens, proliferation of Th1 and B cells, and production of IFN-*γ*, TNF-*α*, IL-1, lL-3, GM-CSF, COX-2, and MMPs and promotes the production of IL-1R*α*, soluble tumor necrosis factor receptor, and TIMPs.
IL-13	B cell, T cell, and macrophage	B cell, monocyte, and endothelial cell	None	Inhibits ADCC and production of inflammatory cytokines (IL-1, IL-6, IL-8, and TNF-*α*), induces expression of IL-1Ra, and promotes B cell differentiation and antibody synthesis.
IL-25 (also called as IL-17E)	Th17 cell, Th2 cell, mast cell, epithelial cell, and macrophage	Th17 cell, NK cell, type 2 myeloid cell, Th9 cell, basophil, eosinophil, mast cell, endothelial cell, macrophage, dendritic cell, and CD4^+^ T cell	None	Inhibits IL-17A and IFN-*γ* production and induces the expression of various chemokines and cytokines (IL-4, IL-5, and IL-13). Expression of IL-25 in articular cartilage inhibits the synthesis of cartilage and bone stimulates the release of NO and IL-6 production.

Cytokine with dual effects				
TGF-*β*	FLS, macrophage, monocyte, NK cell, and T cell	Fibroblast, epithelial cell, Th17 cell, Treg cell, and macrophage	None	Proinflammatory: chemotactic to fibroblasts, promotes fibroblast proliferation, epithelial cell differentiation, and the stability of Th17 cells.
				Anti-inflammatory: in the absence of IL-6, promotes the differentiation and proliferation of Treg cells and inhibits activation of IFN-*γ*, TNF-*α*, macrophages, and antibody production.

Abbreviations: ADCC: antibody-dependent cellular cytotoxicity; APC: antigen-presenting cells; COX-2: cyclooxygenase-2; CXCL: chemokine (C-X-C motif) ligand; FLS: fibroblast-like synoviocytes; GCP-2: granulocyte chemotactic protein-2; GM-CSF: granulocyte-macrophage colony-stimulating factor; IFN: interferon; MHC: major histocompatibility complex; MCP-1: monocyte chemoattractant protein-1; MIC: macrophage inhibitory cytokine; MMP: matrix metalloproteinase; NO: nitric oxide; OPG: osteoprotegerin; PGE2: prostaglandin E2; RANK: receptor activator of nuclear factor-*κ*B; RANKL: receptor activator of nuclear factor kappa *Β* ligand; TIMP: tissue inhibitors of metalloproteinase; TNF: tumor necrosis factor.

**Table 2:** Various chemokines in RA development.

Members	Sources	Ligand(s)	Target(s)	Major functions
CXC				
CXCL1/gro*α*	FLS, chondrocyte, macrophage, endothelial cell, synovial lining cell, and osteoclast	CXCR2, DARC	Neutrophil, macrophage, and fibroblasts	Enhances collagen deposition on RA fibroblasts, thereby fibrillating the synovium.
CXCL4/PF4	Macrophage, monocyte, and T cell	CXCR3	Neutrophil, fibroblast, monocyte, and Th17 cell	Prevents monocyte apoptosis, induces differentiation of macrophages and enhances monocyte phagocytosis and oxygen radical production, exacerbates synovial inflammation, and promotes its chronicity by attracting and activating monocytes to the inflamed tissue.
CXCL5/ENA-78	Monocyte, fibroblast, synovial lining cell, macrophage, and endothelial cell	CXCR2, DARC	Neutrophil, Th17 cell, and FLS	Potent chemotactic factor for neutrophils and also an angiogenic factor. Increased levels of CXCL5 contribute to enhanced levels of RANKL expression.
CXCL6/GCP-2	Fibroblast, endothelial cell	CXCR2	Neutrophil, vascular endothelial cell, and chondrocyte	Plays an important role in neutrophil migration and angiogenesis, promotes angiogenesis, and aggravates joint inflammation.
CXCL7/CTAP-III	Macrophage, monocyte	CXCR2	Neutrophil, connective tissue cell	It has an angiogenic effect that affects many aspects of connective tissue metabolism, including the proliferation of synovial fibroblasts and stimulation of synovial fibrosis.
CXCL8/IL-8	Synoviocyte, macrophage, synovial lining cell, endothelial cell, and monocyte	CXCR1, CXCR2, and DARC	Neutrophil, osteoblast, NK cell, T cell, synovial cell, and chondrocyte, macrophage	Regulates leukocyte adhesion molecule expression and acts as a mediator of angiogenesis.
CXCL9/Mig	Synoviocyte, macrophage, neutrophil, monocyte, endothelial cell, fibroblast, and keratinocyte	CXCR3	T cell, eosinophil, monocyte, and NK cell	Promotes recruitment of activated T lymphocytes and mast cell precursors and their migration to the site of inflammation.
CXCL10/IP-10	Synoviocyte, leukocyte, FLS, chondrocyte, neutrophil, monocyte, endothelial cell, fibroblast, and keratinocyte	CXCR3	Macrophage, FLS, T cell, eosinophil, monocyte, and NK cell	Induces osteoclast differentiation and mediates RANKL expression in synovial CD4^+^ T cells.
CXCL12/SDF-1	Synoviocyte, endothelial cell, osteoblast, and stromal cell	CXCR4	T cell, monocyte, endothelial cell, osteoclast, and stromal cell	Involved in the recruitment of CD4^+^ T cells to the RA synovium, synovial inflammation, and synovial lymphoid neogenesis. It binds to endoglin, which mediates the development of integrin-dependent leukocyte transendothelial migration, osteoclastogenesis, and bone erosion, leading to radiological progression of RA.
CXCL13/BLC	Fibroblast, endothelial cell, follicular dendritic cell, CD4^+^ T cell, monocyte, and macrophage	CXCR5	B cell, Tfh cell, Th17 cell, and Treg cell	Coordinates the migration and preferential sequestration of B and T cells in the inflammatory areas.
CXCL16	Macrophage, fibroblast, dendritic cell, monocyte, B cell, T cell, smooth muscle cell, and endothelial cell	CXCR6	T cells, NK cell, B cell, FLS, plasma cell, and APC	Mediates leukocyte infiltration into synovial tissue involving the MAP kinase (MAPK) pathway and also in lymphocyte recruitment and lymph node organization.

CC				
CCL2/MCP-1	Synoviocyte, fibroblast, macrophage, and osteoblast	CCR2, CCR10,	Monocyte, T cell, NK cell, basophil, macrophage, and osteoclast	Induces angiogenesis, recruits macrophages to the joints, and involves in TNF-mediated osteoclast differentiation.
CCL3/MIP-1*α*	Synoviocyte, monocyte, fibroblast, FLS, neutrophil, macrophage, lymphocyte, basophil, mast cell, and dendritic cell	CCR1, CCR5, and CCR3	Monocyte, T cell, B cell, NK cell, basophil, eosinophil, and dendritic cell	Induces leukocyte chemotaxis, promotes the circulation of T cells into the inflammatory tissues, activates proinflammatory cytokines and RANKL by modulating activation of the PI3K/AKT signaling pathway, and upregulates CD4^+^ T cells.
CCL5/RANTES	Fibroblast, T cell, endothelial cell, chondrocyte, monocyte, and macrophage	CCR1, CCR3, and CCR5	Monocyte, T cell, NK cell, eosinophil, and basophil	Induces MMP-1 and MMP-13 expression as well as collagenase activity by activating RA synovial fibroblasts and may promote IL-1*β*-induced bone destruction.
CCL13/MCP-4	Chondrocyte, fibroblast, epithelial cell, and endothelial cell	CCR2, CCR3	Eosinophil, monocyte, T cell, FLS, and basophil	Stimulates proliferation of synovial fibroblasts.
CCL18/PARC	Neutrophil, dendritic cell	Unknown	T cell, APC, B cell, and macrophage	Promotes the recruitment of T cells by APC.
CCL20/MIP-3*α*	Synovial lining cell, monocyte	CCR6	Th17 cell, B cell, monocyte, dendritic cell, osteoblast, and osteoclast	Recruits IL-17-producing CCR6^+^ Th17 cells into the synovium and promotes osteoblast proliferation and osteoclast differentiation and may cooperate with the RANKL to affect bone formation and resorption.
CCL25/TECK	Dendritic cell, epithelial cell, endothelial cell, and macrophage	CCR9	Macrophage, monocyte, and T cell	CCL25 may be involved in the differentiation of monocytes into macrophages, especially in RA.

C				
XCL1	CD8^+^ T cell, CD4^+^ Th1 cell, and NK cell	XCR1	Monocyte, FLS, T cell, thymocyte, B cell, NK cell, neutrophil, and CD8^+^ dendritic cell	Promotes T cell chemotaxis, stimulates the accumulation of T cells in arthritis joints, and downregulates production of MMP-2 by synovial fibroblasts.

CX_3_C				
CX_3_CL1/Fractalkine	Monocyte, macrophage, FLS, endothelial cell, and dendritic cell	CX_3_CR1	T cell, CD16^+^ monocyte, macrophage, FLS, and osteoclast	Mediates angiogenesis, enhances the adhesion of senescent T cells to synovial fibroblasts, costimulates the production of proinflammatory cytokines, and regulates cytoskeletal structure, proliferation, and migration of synovial fibroblasts.

Abbreviations: APC: antigen-presenting cell; BLC: B lymphocyte chemoattractant; CTAP-III: connective tissue activating protein-III; CX3CL1: Fractalkine; CXCR: CXC chemokine receptor; DARC: duffy antigen receptor for chemokines; ENA-78: epithelial-neutrophil activating protein-78; FLS: fibroblast-like synoviocytes; GCP-2: granulocyte chemotactic protein-2; gro*α*: growth-related gene product *α*; IL-8: interleukin-8; IP-10: interferon-*γ*-inducible protein; Mig: monokine induced by IFN-*γ*; MAPK: mitogen-activated protein kinase; MCP: monocyte chemoattractant protein; MIP: macrophage inflammatory protein; PARC: pulmonary- and activation-regulated chemokine; PF4: platelet factor 4; RANKL: receptor activator of nuclear factor kappa *Β* ligand; RANTES: regulated upon activation normally T cell expressed and secreted; SDF-1: stromal cell-derived factor; TECK: monocyte differentiation and chemotaxis to chemokine ligand 25; Tfh cell: follicular helper T cell; XCL1: lymphotactin.

**Table 3:** MMPs, ADAMTS, and TIMPs in arthritis pathogenesis.

Enzymes/inhibitors	Category	Enzymes	Sources	Targets	Major functions
Metalloproteinases					
MMP-1	Collagenases	Collagenase-1, interstitial collagenase	Monocyte, fibroblast, smooth muscle cell, chondrocyte, macrophage, endothelial cell, and keratinocyte	Collagens (I–III, VII, VIII, and X), gelatin, aggrecan, L-selectin, IL-1*β*, entactin, ovostatin, MMP-2, and MMP-9	Releases MMP-9, promotes Akt dephosphorylation, degrades collagens I, II, II, III, VI, and IX and proteoglycans [66, 67].
MMP-2	Gelatinases	Gelatinase-A, 72 KDa gelatinase	Synoviocyte, CD34^+^ vascular endothelial cells, CD68^+^ macrophage, CD14^+^ monocyte, and stromal cell	Gelatin, collagens I, III, IV–VII, IX and X, Laminin, elastin, Fibronectin, proteoglycan, pro-MMP-13	Increases VEGF expression and angiogenesis, promotes angiogenesis and directs degradation of cartilage matrix.
MMP-3	Stromelysins	Stromelysin-1	FLS, chondrocyte, synovial lining cell, endothelial cell	Collagens (III–V and IX), gelatin, aggrecan, perlecan, decorin, laminin, elastin, casein, osteonectin, ovostatin, entactin, plasminogen, MBP, IL-1*β*, MMP-2, TIMP-2, MMP-7, MMP-8, MMP-9, MMP-13, proteoglycans, and fibronectin	Degrades cartilage connexin, fibronectin, and major components of cartilage such as collagen type IV, VII, IX, and XI, activates collagenase secreted by synoviocytes, directly destroys cartilage tissue, activates interstitial collagenase, and promotes the formation of vasospasm [68].
MMP-8	Collagenases	Collagenase-2, neutrophil collagenase	Neutrophil, plasma cell, fibroblast, dentin cell, endothelial cell, bronchial epithelial cell, keratinocyte, and macrophage	Collagens (I–III, V, VII, VIII, and X), gelatin, aggrecan, and fibronectin	Degrades type I, II, and III collagens, activates MMP-2 and MMP-3, regulates the activity of TNF-*α*, IL-1*β*, and T cell membrane proteins CD2, CD4, and CD8.
MMP-9	Gelatinases	Gelatinase-B, 92 kDa gelatinase	Monocyte, synoviocyte, CD34^+^ vascular endothelial cell, CD68^+^ macrophage, and neutrophil	Collagens (IV, V, VII, X, and XIV), gelatin, entactin, aggrecan, elastin, fibronectin, osteonectin, plasminogen, MBP, and IL-1*β*	Participates in the degradation of ECM including types IV and V collagen, proteoglycans, elastin, and gelatin and in ECM remodeling.
MMP-10	Stromelysins	Stromelysin-2	FLS, chondrocyte, and osteoblast,	Collagens (III–V), gelatin, casein, aggrecan, elastin, MMP-1, and MMP-8	Activates MMP-1, MMP-7, MMP-8, and MMP-9 and 13 prototypes, enhances the dissolution of cartilage collagen by activating procollagenases, and enhances cartilage collagen lysis caused by IL-1 and oncostatin M.
MMP-12	Other enzymes	Macrophage metalloelastase	Macrophage, monocyte, and chondrocyte	Collagen IV, gelatin, elastin, casein, fibronectin, vitronectin, laminin, entactin, fibrinogen, fibrin, and plasminogen	Degrades type IV collagen, fibronectin, laminin, vitronectin, proteoglycan, chondroitin sulfate, and myelin basic protein and activates MMP-2 and MMP-3.
MMP-13 (interstitial collagenase)	Collagenases	Collagenase-3	Chondrocyte, FLS, and macrophage	Collagens (I–IV, IX, X, and XIV), gelatin, plasminogen, aggrecan, perlecan, fibronectin, osteonectin, and MMP-9	Degrades collagen fibers of types I, II, III, V, and XI as well as basement membrane proteoglycans.
MMP-14	MT-MMP	MT1-MMP, MT-MMP-1	Macrophage, myeloid cell, FLS, and CD68^+^ osteoclast	Collagens (I–III), gelatin, casein, fibronectin, laminin, vitronectin, entactin, proteoglycans, and MMP-2 and 13	Promotes FLS to invade cartilage. MT1-MMP degrades collagen types I, II, and III, laminin-1 and laminin-5, fibronectin, vitronectin, fibrin, and aggrecan, and activates pro-MMP-2 and pro-MMP-13 on the cell surface [69, 70].

ADAMTS [71, 72]					
ADAMTS-1	Aggrecanases	METH-1, Aggrecanase-3	Chondrocyte, macrophage	Aggrecan, versican	Cleaves the proteoglycan versican.
ADAMTS-4	Aggrecanases	Aggrecanase-1	FLS, chondrocyte	Aggrecan, crevican, COMP, decorin, fibromodulin, and versican	Cleaves aggrecan.
ADAMTS-5	Aggrecanases	Aggrecanase-2, ADAMTS-11	FLS, stromal cell	Aggrecan	Cleaves aggrecan.
ADAMTS-7	None	ADAMTS-7B	FLS, chondrocyte	COMP, *α*_2_M	Degrades cartilage oligomeric matrix protein (COMP).
ADAMTS-9	None	KIAA1312	FLS	Aggrecan, versican	Degrades aggrecan and has the potential to cleave other cartilage molecules.
ADAMTS-12	None	None	FLS, chondrocyte	Aggrecan, COMP, and *α*_2_M	Degrades COMP.

Inhibitors					
TIMP-1	Glycoprotein	None	Macrophage, connective tissue cell, chondrocyte, FLS, T cell, and monocyte	MMP-3, MMP-9, MMP-14, MMP-16, MMP-19, MMP-24, ADAM10, and pro-MMP-9	Weak inhibition of MMP-14, MMP-16, MMP-19, and MMP-24 and ADAM10. Inhibits pro-MMP interactions with pro-MMP-9, formation of synovial blood vessels, activation of MMP-3 and 9, and synovial vascular invasion in RA.
TIMP-2	Glycoprotein	None	Chondrocyte, FLS, T cell, and monocyte	ADAM12, pro-MMP-2, and MMP-9	Inhibits all the MMPs (prevents overactivation of MMP-9), ADAM12, and pro-MMP interactions with pro-MMP-2.
TIMP-3	Glycoprotein	None	FLS, chondrocyte, macrophage, and monocyte	ADAM10, ADAM12, ADAM17, ADAM28, and ADAM33; ADAMTS-1-2 and 4-5; pro-MMP-2,9; and MMP-2, MMP-9, and MMP-13	Inhibits all the MMPs and ADAM10, ADAM12, ADAM17, ADAM28, and ADAM33; ADAMTS-1, ADAMTS-4, and ADAMTS-5, ADAMTS-2 (weak); and pro-MMP interactions with pro-MMP-9 and pro-MMP-2.
TIMP-4	Glycoprotein	None	FLS, endothelial cell, T cell, and monocyte (less)	ADAM17d, ADAM28, and ADAM33 and pro-MMP-2	Inhibits most of the MMPs and ADAM17d and ADAM28, ADAM33 (weak), pro-MMP interactions with pro-MMP-2) and development of arthritis.

Abbreviations: ADAMTS: a disintegrin and metalloproteinase with thrombospondin motifs; COMP: cartilage oligomeric matrix protein; GEP: granulin-epithelin precursor; TIMP: tissue inhibitor of metalloproteinases; vWFCP: von Willebrand factor cleaving protease; *α*2M: *α*2-macroglobulin.

## Additional Points

*Key Messages*. Rheumatoid arthritis is a multi-factorial syndrome involving interactions between genetic, epigenetic and environmental factors. Initiation events damaging joints start very early during the autoimmune phase of arthritis development. Comprehensive knowledge on pathogenesis is a prerequisite for developing optimal treatment and potential drugs. Targeting a single effector molecule is insufficient to block cartilage and bone damage in arthritis. Since rheumatoid arthritis is an immune-mediated disorder, therapeutics restoring immune balance can improve clinical therapy.
